# Prediction of transient and permanent protein interactions using AI methods

**DOI:** 10.6026/97320630019749

**Published:** 2023-06-30

**Authors:** Kiran Kumar A, Syed Mohammad Shayez Karim, Mayank Kumar, Rathore Ravindranath Singh

**Affiliations:** 1 Department of Bioinformatics, Central University of South Bihar, Gaya, Bihar-824236, India

**Keywords:** Transient and Permanent Protein-Protein Interactions, Machine Learning, *Scikit-learn*, Deep Learning, *Tensor Flow*.

## Abstract

Protein-protein interactions (PPIs) can be classified as permanent or transient interactions based on their stability or lifetime. Understanding the precise details of such protein interactions will pave the way for the discovery of inhibitors and for understanding the nature and function of PPIs. In the present work, 43 relevant physicochemical, geometrical and structural features were calculated for a curated dataset from the literature, comprising of 402 protein-protein complexes of permanent and transient categories, and 5 different Supervised Machine Learning models were developed with *Scikit-learn* to predict transient and permanent PPI. Additionally, deep learning method with Artificial Neural Network was also performed using *Tensor Flow* and *Keras*. Predicted models achieved accuracy ranging from 76.54% to 82.71% and k-NN has achieved the highest accuracy. Detailed analysis of these methods revealed that Interface areas such as Percent interface accessible area, Interface accessible area and Total interface area and the parameters defining the shape of the PPI interface such as Planarity, Eccentricity and Circularity are the most discriminating factors between these two categories. The present method could serve as an effective tool to understand the mechanism of protein association and to predict the transient and permanent interactions, which could supplement the costly and time-consuming experimental techniques.

## Background:

A host of biological and cellular activities, such as gene replication, transcription, translation, cell cycle regulation, signal transmission, and immune response, rely on protein-protein interactions. Protein-protein interactions (PPIs) are vital for understanding how proteins work together in the cell to accomplish biological tasks in a coordinated manner [1, 2]. An estimated 130,000 to 650,000 different types of protein-protein interactions exist in human cells [3, 4, 5]. Such interactions belong to permanent or transient categories of interactions, which play a specific role in cellular activities [6, 7]. Permanent complexes such as enzyme-inhibitor, antigen-antibody, and oligomeric enzyme are composed of proteins that bind tightly and permanently, whereas transient complexes weakly associate and form just temporarily to produce specific effects like signal transduction, disease related pathways and cell cycle [8, 9]. These interactions are distinguished by their dissociation constant (Kd) as permanent complexes having dissociation value in the nM range (1 x 10-9 M) or lower [10, 11], whereas transient complexes have dissociation constant in the µM range or higher (1 x 10-6 M) [12, 13, 14]. The ability to manipulate these protein-protein interactions could be useful in the development of PPI modulators, which could open up new avenues for biologics research [15, 16]. A deep structural understanding of such complexes at the atomic level will enhance our knowledge of biological processes and may facilitate biomedical and biotechnological interventions easier. Earlier, investigations have been carried out primarily using sequence-based features [17, 18, 19, 20] to elucidate the differences between permanent and transient protein interactions. Permanent interaction sites have been found to possess more hydrophobic residues, more conserved, and their interfaces contain fewer gaps in multiple sequence alignments of protein families. On the other hand, transient interfaces have more polar residues, and they form smaller interfaces than permanent interfaces [19]. Machine-learning techniques have proven to be effective in predicting and distinguishing different types of PPIs [21, 22, 23]. Recently, a wide number of state-of-the-art techniques to predict protein-protein interactions have been reviewed [24]. In the present study, we have employed several supervised machine learning and deep learning methods to classify transient and permanent interactions by calculating various physicochemical, geometrical and structural factors that define transient and permanent protein interactions. In our calculations, different properties like Percent interface accessible area, Interface accessible area, and Total interface area, Planarity, Circularity and Eccentricity were discovered to be capable of discriminating between transient and permanent protein interactions. Our approaches of diverse supervised machine learning algorithms and Artificial Neural Networks (ANN) were able to differentiate 402 protein-protein complexes with an accuracy of 76.54 to 82.71%.

##  Materials and methods:

## Dataset preparation and processing:

**Figure 1:**
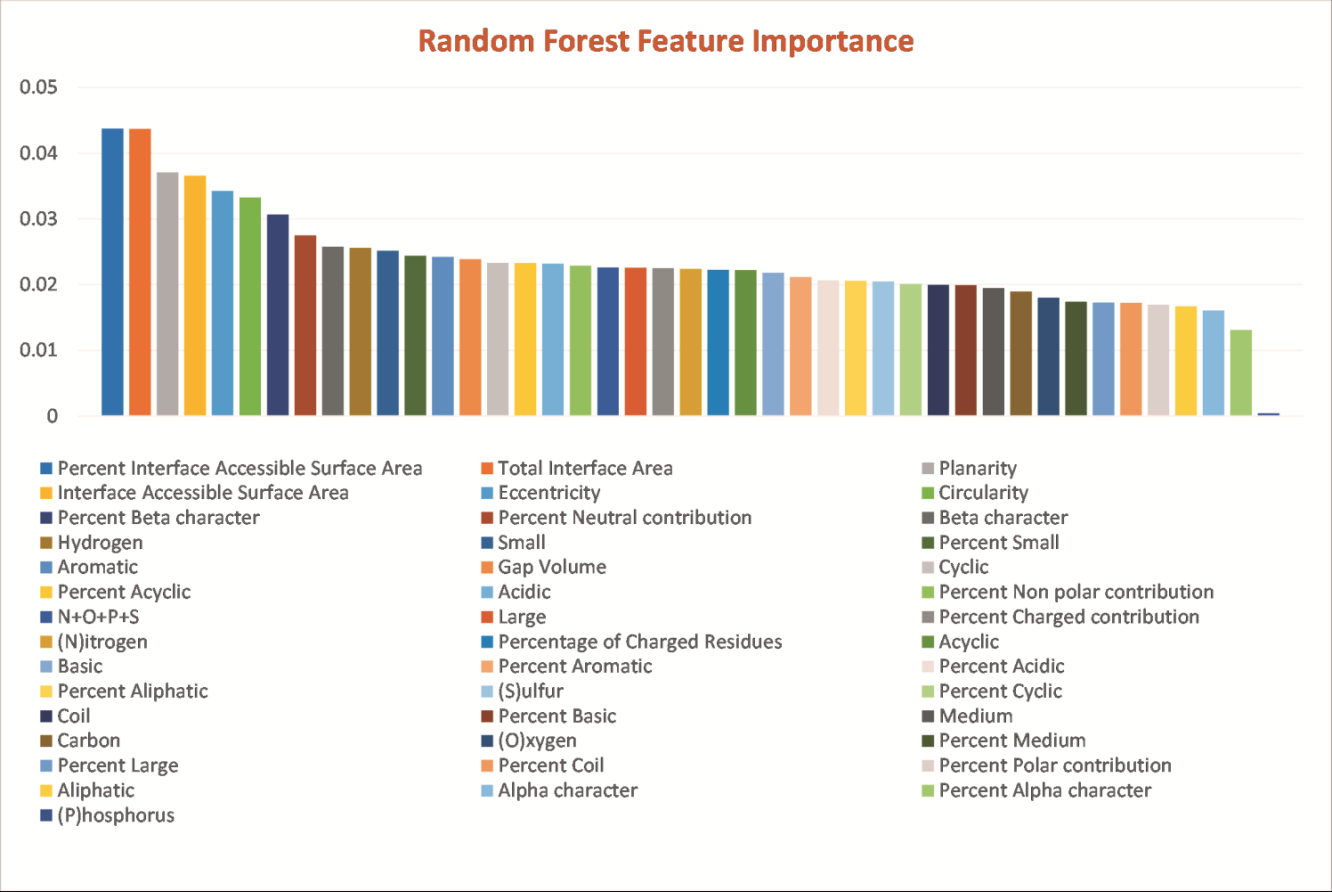
Feature importance plot performed with Random Forest. Feature significance score is displayed on Y-axis. Definition of features is as per reference [25]

Dataset of protein complexes to study transient and permanent interactions were compiled from the literature [19, 20, 21]. The dataset contains a total of 402 transient and permanent protein complexes containing 201 complexes belonging to each category (List of PDB entries included in Supplementary Table S1 - see PDF).Various categories of structural, physicochemical and geometrical descriptors were calculated using 2P2I inspector [25]. We have calculated a total of 43 different features such as total interface area, gap volume, percent interface accessible surface area, neutral/polar/nonpolar contribution, planarity, circularity, eccentricity and others (listed in Figure 1). Missing data and outliers were cleaned and data were pre-processed using *Scikit-learn* Standard Scaler utility. All descriptors were rescaled between 0 and 1.

## Supervised Machine Learning with Python:

*Scikit-learn* was used to construct the classification models and training the data to determine the best parameters for the training model using different algorithms such as k-Nearest Neighbour (k-NN) [26], Logistic Regression [27], Decision Tree [28], Random Forest [29] and Support Vector Machine (SVM) [30]. *Pandas v1.1.5*, *matplotlib v3.2.2*, *NumPy v1.19.5*, *SciPy v1.4.1*, *Scikit-learn v0.22.2* [31], and *seaborn v0.11.2* were used to perform the machine learning. In all our models, the datasets were divided into training and test sets, in the ratio of 80:20. In k-NN, several distance metrics were evaluated in *Scikit-learn*, including k = 1 to 5 nearest neighbours, to predict the data. In Random Forest, the number of decision trees was set as 500. For Logistic Regression, different logistic regression classifiers have been employed by varying C value from 100 to 1000 and the best accuracy was achieved with C=500. The precision score, sensitivity or recall and F1 score, which is the weighted average of both the precision score and recall were calculated for each algorithm (detail description about these parameters provided in the supplementary material). These performance measurements were calculated for each class that is transient and permanent and the geometric mean (G-mean) of sensitivity and specificity was also computed (Table 1). We performed variable importance calculation using Boruta and Random Forest in Python as shown in the (Figure 1).

##  Deep Learning with Tensor Flow:

We used *Tensor Flow* and *Keras* to implement the deep learning. Deep learning models [32] are made up of multiple computational layers that process the input in a hierarchical manner. Each layer takes an input and outputs a non-linear function of a weighted linear combination of the input values. A deep architecture is created when the output of one processing layer becomes an input to the next processing layer. Networks with two hidden layers were adopted to compare their performance in our study. We used ReLU as an activation function for the two hidden layers and sigmoid function for the output layer. As earlier, the data were divided into training and test set in 80:20 ratios.

## Results and discussion:

Based on 43 descriptors, several machine learning and deep learning methods were attempted to arrive at consensus results. The accuracy of the methods and other performance evaluation metrics were calculated and reported in Table 1. The accuracy of different methods achieved, range between 76.54% to 82.71% prediction of the data using physicochemical, geometrical and structural features. The highest accuracy of 82.71% was achieved with k-NN (Table 1). The values of precision and F1 score of the method were 0.827 and 0.826, respectively. The other supervised machine learning algorithms - Random Forest, Logistic Regression, Decision Trees and SVM have yielded accuracies of 81.48%, 80.24%, 77.77% and 76.54%, respectively. The deep learning with ANN achieved the accuracy of 79% with 500 epochs and with *adam* as the optimizer for 43 input dimensions. To elucidate the relative feature importance in transient and permanent categories, the feature contributions were also calculated. One of the most discriminating category of features in this classification procedure is interface areas, namely, Percent interface accessible area, Interface accessible area and Total interface area with feature importance score of 0.0437, 0.0436 and 0.036, respectively. The value of these parameters for transient PPI have been observed significantly lower as compared to permanent PPI. The average value of Percent interface accessible area, Total interface area and Interface accessible area for transient PPI category have been observed to be 10.98%, 2594.4Å² and1291.2Å², respectively, as against 15.11%, 3819.1Å² and1911.5Å², respectively for in permanent PPI category.

The second most important category of discriminating features is the one that describe the shape of interface such as Planarity, Eccentricity and Circularity with feature importance scores of 0.037, 0.034 and0.033, respectively. The Planarity describes the rough or bent interface [25, 33] and calculated as root mean square deviation (RMSD) for all interface atoms from the best fitted least square plane of all the interface atoms. The average planarity coefficient in transient PPI category varies between 0.29-7.2 Å (Avg. 3.02 Å) as compared to 0.57-10.6 Å (Avg. 3.8 Å) in permanent PPI category. Eccentricity (roundness of the interface and opposite to the curvature) suggest slightly low curvature in transient category, 0.2-0.99 (Avg. 0.73) than in permanent PPI, 0.12-0.979 (Avg. 0.68). Another such measure i.e., Circularity coefficient is also found to be slightly lower that varies between 0.123-0.98 (Avg. 0.61) in transient PPI than 0.20-0.99 (Avg. 0.68) in permanent PPI category. A related parameter of interface shape is the Gap volume with a feature importance score of 0.023. In transient categories the average gap volume was slightly higher 7775.2 Å³ as compared to permanent category having a value of 7717.1 Å³. The third most categories of discriminating features are the percentage of beta character with a feature important score of 0.031. For transient PPI its value varies in between 0-100 (Avg. 21.6), and in permanent PPI the value is higher, which has been found to be 0-94 (Avg. 26.6).

## Conclusion:

Transient and permanent protein-protein interactions are significant in many biological processes. In the present work, we used a dataset, compiled from the literature and extracted physicochemical, geometrical and structural features from each of the 201 permanent and transient protein-protein complexes. Interface areas, shape of the interface and percent beta character are the three distinct categories of features, which prominently discriminate transient and permanent interactions. The method we proposed here could be useful in engineering permanent or transient PPIs, notably in the conversion of permanent docking interfaces to transient docking interfaces or vice versa using interface mutations [16]. The ability to manipulate these protein-protein interactions should aid in structure-aided biologics discovery. In addition, the present methodology may also be used to classify other similar types of interactions such as protein-DNA and protein-RNA interactions.

## Associated Data:

Supplementary Materials

## Figures and Tables

**Table 1:** Performance measurements of Machine learning models obtained With Scikit-learn.

**ML Method**	**Accuracy**	**Class**	**Sensitivity**	**G-Mean of Sensitivity and Specificity**	**Precision**	**F1 Score**
k-NN		Transient	0.804	0.804	0.846	0.824
		Permanent	0.85	0.804	0.809	0.828
	82.71	Average	0.827	0.804	0.827	0.826
Random		Transient	0.756	0.813	0.861	0.805
Forest		Permanent	0.875	0.813	0.778	0.823
	81.48	Average	0.815	0.813	0.819	0.814
Logistic		Transient	0.804	0.801	0.804	0.804
Regression		Permanent	0.8	0.801	0.8	0.8
	80.24	Average	0.802	0.801	0.802	0.802
Decision		Transient	0.75	0.776	0.789	0.769
Tree		Permanent	0.804	0.776	0.767	0.785
	77.77	Average	0.777	0.776	0.778	0.779
SVM		Transient	0.744	0.766	0.8	0.77
	76.54	Permanent	0.789	0.766	0.731	0.758
		Average	0.766	0.766	0.765	0.764
